# Neutrophilia, gelatinase release and microvascular leakage induced by human mast cell tryptase in a mouse model: Lack of a role of protease‐activated receptor 2 (PAR2)

**DOI:** 10.1111/cea.13108

**Published:** 2018-04-27

**Authors:** M. E. M. S. Khedr, A. M. Abdelmotelb, S. L. F. Pender, X. Zhou, A. F. Walls

**Affiliations:** ^1^ Clinical and Experimental Sciences Academic Unit Faculty of Medicine University of Southampton Southampton UK; ^2^ Faculty of Medicine Suez Canal University Ismailia Egypt; ^3^ Faculty of Medicine Tanta University Tanta Egypt

**Keywords:** allergy, inflammation, mast cells/basophils, neutrophils, transgenic/knockout mice

## Abstract

**Background:**

Tryptase, the most abundant protease of the human mast cell, has been implicated as a key mediator of allergic inflammation that acts through activation of PAR2.

**Objectives:**

To investigate the contribution of PAR2 in the pro‐inflammatory actions mediated by tryptase in a mice model.

**Methods:**

We have injected recombinant human βII‐tryptase into the peritoneum of PAR2‐deficient and wild‐type C57BL/6 mice. After 6, 12 and 24 hours, mice were killed, peritoneal lavage performed and inflammatory changes investigated.

**Results:**

Tryptase stimulated an increase in neutrophil numbers in the peritoneum, but responses did not differ between PAR2‐deficient and wild‐type mice. Heat inactivation of tryptase or pre‐incubation with a selective tryptase inhibitor reduced neutrophilia, but neutrophil accumulation was not elicited with a peptide agonist of PAR2 (SLIGRL‐NH_2_). Zymography indicated that tryptase stimulated the release of matrix metalloproteinases (MMP) 2 and 9 in the peritoneum of both mouse strains. Studies involving immunomagnetic isolation of neutrophils suggested that neutrophils represent the major cellular source of tryptase‐induced MMP2 and MMP9. At 24 hours after tryptase injection, there was increased microvascular leakage as indicated by high levels of albumin in peritoneal lavage fluid, and this appeared to be partially abolished by heat‐inactivating tryptase or addition of a protease inhibitor. There was no corresponding increase in levels of histamine or total protein. The extent of tryptase‐induced microvascular leakage or gelatinase release into the peritoneum did not differ between PAR2‐deficient and wild‐type mice.

**Conclusions:**

Our findings indicate that tryptase is a potent stimulus for neutrophil accumulation, MMP release and microvascular leakage. Although these actions required an intact catalytic site, the primary mechanism of tryptase in vivo would appear to involve processes independent of PAR2.

## 1 INTRODUCTION

Tryptase, the most abundant product of human mast cells, is emerging as a key mediator of inflammation, as well as being an important marker for mast cell activation in allergic disease. Although relatively few naturally occurring substrates have been identified for this tetrameric serine protease, a range of potent pro‐inflammatory actions has been described for tryptase following its administration into animal models, or when it is transferred to cells or tissues. Reports that tryptase can activate protease‐activated receptor 2 (PAR2)^1^, ^2^, ^3^ have led to suggestions that this G protein‐coupled receptor may represent a key cellular target.^4^, ^5^, ^6^, ^7^ Tryptase can like certain other tryptic proteases cleave PAR2 to expose a “tethered ligand” leading to signal transduction. The idea that PAR2 has a pivotal role in mediating the actions of tryptase has received support from comparisons of the actions of PAR2 agonists with those of tryptase. However, some discrepancies have been reported, raising questions over the degree to which PAR2 activation may be involved.^2^, ^8^, ^9^

Transfer of purified human tryptase into the peritoneum^10^ or trachea of mice,^11^ or the skin of guinea‐pigs^12^ or sheep,^13^ has been found to stimulate massive accumulation of neutrophils or eosinophils, and, in some cases, prolonged microvascular leakage. Peritoneal injection of the mouse tryptase mMCP‐6 is also associated with intraperitoneal neutrophilia,^14^ while injection with mMCP‐7, another mouse tryptase, has induced eosinophil accumulation in the peritoneal cavity.^11^ In all in vivo models studied, the actions of tryptase have been dependent on an intact catalytic site, and inhibitors of tryptase have shown efficacy in models of asthma in sheep,^15^ mice 16 and humans,^17^, ^18^ as well as in a rat model of colitis.^19^ Transfer of peptide agonists of PAR2 such as SLIGRL‐NH_2_ in vivo has also been associated with induction of microvascular leakage and neutrophilia,^20^, ^21^, ^22^ although paradoxically there have been reports also of anti‐inflammatory actions in vivo.^23^

Tryptase has been demonstrated to induce profound changes in the behaviour of various cell types including mast cells, neutrophils, eosinophils, endothelial cells, airway smooth muscles and several cell lines. Tryptase can induce the degranulation of human mast cells^12^ and eosinophils^24^ and induce mitogenic responses and cytokine release from endothelial cells,^25^ epithelial cells^26^ and airway smooth muscle cells.^27^, ^28^ These cell types express PAR2, and in most cases, effects similar to those for tryptase have been found to be elicited also by PAR2 agonists^24^, ^29^, ^30^, ^31^, ^32^; and cell signalling responses described in some cell models have been similar with both tryptase and PAR2 agonists.^33^, ^34^ A lack of effective PAR2 antagonists has hindered their application in such studies, although the peptide antagonist FSLLRY‐NH_2_ has been reported to reverse some of the cell signalling responses of tryptase in cells.^29^, ^35^ There have been relatively few direct comparisons between tryptase and other potential PAR2 agonists, but peptide agonists of PAR2 have failed to elicit certain actions of tryptase such as stimulation of IL‐8 release from airway smooth muscle cells,^36^ or the degranulation of lung mast cells^37^ and eosinophils.^24^

The advent of PAR2 knockout mice with C57BL/6J background (which are apparently phenotypically normal) in which lack of PAR2 functionality has been demonstrated^38^, ^39^, ^40^ has provided evidence for a key role for this receptor in disease. These have included models for studying the spread of melanoma metastases^39^ and joint swelling in arthritis.^38^, ^40^ While intra‐articular injection of tryptase has been reported to stimulate joint swelling and hyperaemia in wild‐type mice, it failed to do so in PAR2 knockout mice suggesting that there is a dependence on PAR2 activation, but detailed investigation of cellular changes or other features of inflammation was not examined.

In this study, we have demonstrated that tryptase is able to stimulate inflammatory cell accumulation, microvascular leakage and gelatinase release in the peritoneum of both wild‐type and PAR2‐deficient mice models. Our findings cast doubt on PAR2 having a key role in mediating these changes.

## 2 MATERIALS AND METHODS

### 2.1 Preparation and purification of tryptase

Tryptase was isolated from a *Pichia pastoris* expression system for βII‐tryptase following protocols similar to those described by Niles et al,^41^ with sequential purification by hydrophobic interaction chromatography followed by heparin affinity chromatography. The initial step involved passing the tryptase‐rich supernatant down a 25 mL butyl Sepharose (GE Healthcare, Amersham) column at 22°C, washing with buffer A (10 mmol L^−1^ MES, 1 mol L^−1^ (NH_4_)_2_SO_4_, 0.5 mol L^−1^ NaCl, 10% (v/v glycerol), pH 6.1) and eluting with buffer B (10 mmol L^−1^ MES, 0.2 mol L^−1^ NaCl, 10% (v/v) glycerol, pH 5.5). Fractions of 5 mL were collected, and tryptase activity was determined using the chromogenic substrate N‐α‐benzoyl‐DL‐arginine p‐nitroanilide hydrochloride (BApNA; vide infra). Tryptase‐rich fractions were passed down a 25 mL heparin‐agarose column (Sigma, Gillingham, UK), washing with buffer B and eluting with a 0.2 to 2 mol L^−1^ NaCl gradient mixing buffer B with 0 to 75% buffer C (10 mmol/L MES, 2 mol L^−1^ NaCl, 10% (v/v) glycerol, pH 6.1). Fractions (5 mL) with high tryptase activity were eluted between 1.04 and 1.29 mol L^−1^ NaCl, and these were concentrated using centrifugal concentrators with 30 kDa cut‐off (Merck Millipore, Watford, UK) and injected into a BioSep‐Sec‐S‐3000 size exclusion column employing an HPLC ICS 3000 pump (Dionex/Thermo Fisher, Sunnyvale, CA). Fractions of 0.5 mL were collected and analysed for tryptase activity.

### 2.2 Characterization of purified tryptase

SDS‐PAGE analysis was performed with a NuPAGE Bis‐Tris 4‐12% gradient gel (Invitrogen/Thermo Fisher, Inchinnan, UK) under reducing conditions, and a single band was observed with a molecular weight of 35 kDa consistent with that of the monomeric form of tryptase. The identity as tryptase was confirmed by Western blotting with the tryptase‐specific monoclonal antibody AA5**.** Endotoxin levels as assayed by the Chromogenic Limulus Amoebocyte Lysate (LAL) Endotoxin Assay Kit, Toxin Sensor™ from GenScript (Piscataway, NJ) were less than 0.08 EU/1U tryptase in all preparations used in the study.

Tryptase activity was measured by determining cleavage of BApNA spectrophotometrically at 410 nm for 10 minutes at 25°C in a Thermomax microplate reader (Molecular Devices, Wokingham, UK) according a procedure described previously (39). The extinction value (ε) of BApNA was taken as 8800 M^−1 ^cm^−1^. A colorimetric protein assay using bicinchoninic acid was employed in accordance with the manufacturer's instructions (Sigma) using bovine serum albumin as standard. The specific activity of the tryptase preparations employed ranged from 9 to 12.2 U mg^−1^, where 1 unit was taken as the amount of tryptase that can cleave BApNA to release 1 μmol nitroanilide per min at 25°C.

### 2.3 Animals

Colonies of mice lacking the PAR2 gene (PAR2^−/−^) and the corresponding wild‐type (PAR^+/+^) were kind gifts from Kowa Company Ltd (Tokyo, Japan). Both colonies were C57BL/6 genetic background and generated as described by Ferrell et al.^42^ The animals were housed and maintained at the University of Strathclyde (courtesy of Professor Robin Plevin) before being transferred to and maintained at the University of Southampton. All of the animals employed weighed between 25 and 35 g and were housed in standard cages in a temperature‐controlled room (from 19 to 24°C) in an SPF environment, with a light cycle of 10 hours off and 14 hours on. Food and tap water were available ad libitum, with stock mice fed RM1 rodent maintenance pellets and breeding mice RM3 rodent breeding pellets (till weaning). Bedding material was sawdust with shredded cardboard. There were no more than five animals per cage. Mice were randomly assigned to each treatment group. Mice from different treatment groups were maintained in the same cage. The studies were conducted in accordance with the Animals (Scientific Procedures) Act 1986 (UK), under project licence PPL30/2564, and experimental protocols were approved by the Home Office UK and the University of Southampton Animal Care and Ethical Review Committee.

Three mice tail biopsy samples from each genotype were subjected to genotyping. Tissues were homogenized using a RiboLyser™ homogenizer (Hybaid Ltd., Ashford, UK) in 1.5 mL Lysing Matrix D tubes (MP Biomedicals, Solon, OH) with TRI Reagent (Sigma). Confirmation of the presence of the gene for PAR2 was confirmed by performing the PCR using the following primers; 5′‐ATGCGAAGTCTCAGCCTGGCG‐3′ and 5′‐GAGAGGAGGTCGGCCAAGGCC‐3′ to yield a 380‐bp PCR product. The PCR was generally performed for 35 cycles with initial denaturation at 95°C for 5 minutes, annealing at 51°C then for 2 cycle/min and extension at 72°C/cycle (3 minutes) and a final extension (last cycle) at 72°C for 10 minutes. The absence of the PAR2 gene was tested by detection of neomycin gene using the following primers; NeoFwr 5′GAGGAAGCGGTCAGCCCATT3′ and NeoRev 3′TCTTCCTATTGACTAAACGG5′ with amplicon size of 281 bp. The PCR was generally for 33 cycles with initial denaturation 95°C for 10 minutes, annealing at 68°C for 1 minutes and 30 seconds extension at 72°C and a final extension (last cycle) at 72°C for 10 minutes. The PCR was concluded by cooling down to 4°C. PCR products were analysed immediately or stored at 4°C until further analysis. PCR products were separated on 2% agarose gel and visualized with ethidium bromide staining.

### 2.4 Injection of mice

Animals were injected i.p. as described previously with 0.5 μg tryptase in 0.5 mL 0.9% saline, a quantity selected as it had provoked extensive cell accumulation in this model.^10^ The vehicle control was 0.9% saline. Other experimental controls consisted of tryptase that had been heat‐inactivated for 20 minutes at 94°C or incubated with 50 μg mL^−1^ with selective tryptase inhibitor A (Sanofi‐Aventis, Bridgewater, NJ; Ki 39 Nm)^43^ for 60 minutes, and the degree of inhibition was assessed by chromogenic substrate BApNA as described above. Also injected was inhibitor A alone or PAR peptide agonist SLIGRL‐NH_2_ (1 μg mL^−1^) or a peptide with the same amino acids but with a “scrambled” sequence (LSIGRL‐NH_2_; 1 μg mL^−1^). Tryptase was handled with care to avoid loss of enzymatic activity and was kept on ice and diluted with saline immediately before injection of the animal. The degree of enzyme inhibition was assessed spectrophotometrically using the chromogenic substrate BApNA. Where inadvertent injection or damage to the gut was suspected, such animals were excluded from the study.

### 2.5 Peritoneal lavage

At 6, 12 and 24 hours following injection, mice were killed by cervical dislocation. After swabbing the abdominal skin with 70% ethanol, a midline incision of the abdomen was made, and 5 ml saline was injected into the peritoneum. Abdominal massage was performed for 30 seconds, and then saline was collected into tubes maintained on ice. Total numbers of nucleated cells were determined using an improved Neubauer haemocytometer with 0.4% Trypan blue. The peritoneal lavage fluid was centrifuged at 300 *g* for 10 minutes at 4°C. The supernatant was aspirated and stored at −20°C.

Cell pellets were resuspended in MEM with 5% FCS, and cell number was adjusted to 10^6^ cell per mL. Cytocentrifuge preparations were made using a Cytospin 3 (Shandon Southern, Runcorn, UK) with 100 μL aliquots of cell suspension, and slides were left to air‐dry overnight. One slide for each sample was stained with eosin/methylene blue stain (Rapid Romanowsky, Raymond A Lamb/Thermo Fisher, Loughborough, UK) according to the manufacturer's instructions. Differential cell counts were performed, counting a minimum of 500 cells per slide in at least 5 fields at a ×1000 magnification using a Standard 20 microscope (Carl Zeiss, Göttingen, Germany). Two researchers were involved in collection of samples to facilitate blinding. The initial code for each was randomly assigned a new code prior to analysis of data, and the code broken on statistical analysis.

### 2.6 Gelatin zymography

Supernatant from peritoneal lavage fluid was added to a non‐reducing sample buffer (4% SDS, 0.125 mol L^−1^ Tris‐HCl pH 6.8, 0.003% bromophenol blue and 20% glycerol) and applied to an 8% polyacrylamide gel containing 1 mg mL^−1^ gelatin. After electrophoresis, gels were incubated overnight at 37°C in MMP proteolysis buffer (50 mmol L^−1^ Tris‐HCl pH 7.8, 0.5 mmol L^−1^ NaCl and 50 mmol L^−1^ CaCl_2_) and then stained with Coomassie blue dye. Gels were photographed using a Molecular Imager GS‐800 calibrated densitometer (Bio‐Rad Laboratories, Hemel Hempstead, UK), and intensity and size of the bands were determined relative to a positive control for MMP2 and MMP9 activity; a supernatant from the human fibrosarcoma cell line (HT1080) was employed.^44^

### 2.7 Immunomagnetic purification of neutrophils

In order to examine the potential contribution of neutrophils to the response of mice to tryptase, peritoneal cells were collected from naїve mice and from mice which had been injected intraperitoneally with tryptase (0.5 μg/mouse) or casein, an established stimulus for neutrophil accumulation, (0.5 mL of 9% solution/mouse).^45^ The casein for injection was prepared as described previously^46^ with 9% casein hydrolysate in PBS pH 7.2 containing 0.9 mmol L^−1^ CaCl_2_ and 0.5 mmol L^−1^ MgCl_2_. After 24 hours, peritoneal lavage was performed as described above.

Cells were passed through nylon mesh (30 μm, BD Falcon, Erembodegem, Belgium), cell numbers were determined, and the cell suspension was centrifuged at 300 g for 10 minutes at 4°C. Cells were resuspended in 200 μL buffer (0.5% BSA, 2 mmol L^−1^ EDTA in PBS, pH 7.2). Biotinylated antilymphocyte antigen 6G (LY‐6G; Miltenyi Biotec, Bisley, UK) was added, mixed well and incubated for 10 minutes at 4°C. A 150 μL aliquot of the buffer was mixed well with antibiotin MicroBeads (Miltenyi Biotec) and incubated for 15 minutes at 4°C. Cells were washed and resuspended in the buffer. Labelled cells were collected using a ferromagnetic column (LS column, Miltenyi Biotec), cell number was determined, and the cell suspension was resuspended in RPMI 1640 conditioned medium and seeded at a density of 10^6^ cells/well in 24‐well tissue culture plates (Greiner Bio‐One; Stonehouse, UK). Sorted cell populations were treated with tryptase or other compounds, and at 1, 6 or 24 hours, supernatants were collected for gelatin zymography.

### 2.8 Assays for markers in peritoneal lavage fluid

Quantitative colorimetric albumin bromocresol green (BCG) assay kits (QuantiChrom, BioAssay Systems, Hayward, CA) with a detection range from 0.1 mg mL^−1^ to 50 mg mL^−1^ were employed measuring the optical density over the range 570‐670 nm in a Thermomax plate reader (Molecular Devices). Total protein concentrations were determined using the bicinchoninic acid method referred to above. An automated glass fibre‐based technique was employed to determine levels of histamine in peritoneal lavage fluid where samples loaded to pre‐coated FiberPlate assay plates (RefLab, Copenhagen, Denmark). Piperazine‐1, 4‐bis (2‐ethanesulphonic acid) (PIPES) buffer was added, plates were incubated for 60 minutes at 37°C and, after washing shipped to RefLab, Denmark, for analysis. Elastin zymography was carried out according to the procedure described by Forough et al^47^ with samples electrophoresed on a 10%‐12% SDS‐polyacrylamide gel with 1 mg mL^−1^ soluble κ‐elastin under non‐reducing conditions and porcine pancreatic elastase type II‐A as standard (both Sigma). After electrophoresis, the gels were washed in 2.5% Triton X‐100 for 30 minutes and then incubated at 37°C in 50 mmol L^−1^ Tris buffer, pH 7.8, containing 10 mmol L^−1^ CaCl_2_ for 20 hours prior to staining with 0.002% Coomassie brilliant blue (G250).

### 2.9 Statistics

The Kruskal‐Wallis nonparametric test was used to investigate differences between groups, and if significant, then the Mann‐Whitney *U* test was employed for pre‐planned comparisons between two groups of mice. The degree of association between variables was analysed by calculation of Spearman's coefficient of rank correlation (*r*_s_). IBM SPSS Statistics for Windows, version 24.0 (IBM Corp, Armonk, NY, USA), and GraphPad Prism for Windows, version 7.7.1, GraphPad Software (La Jolla California USA, http://www.graphpad.com) were used for analysis of data and preparation of graphs. *P* < .05 was taken as significant.

## 3 RESULTS

### 3.1 Tryptase‐induced cell accumulation

**Figure 1:**
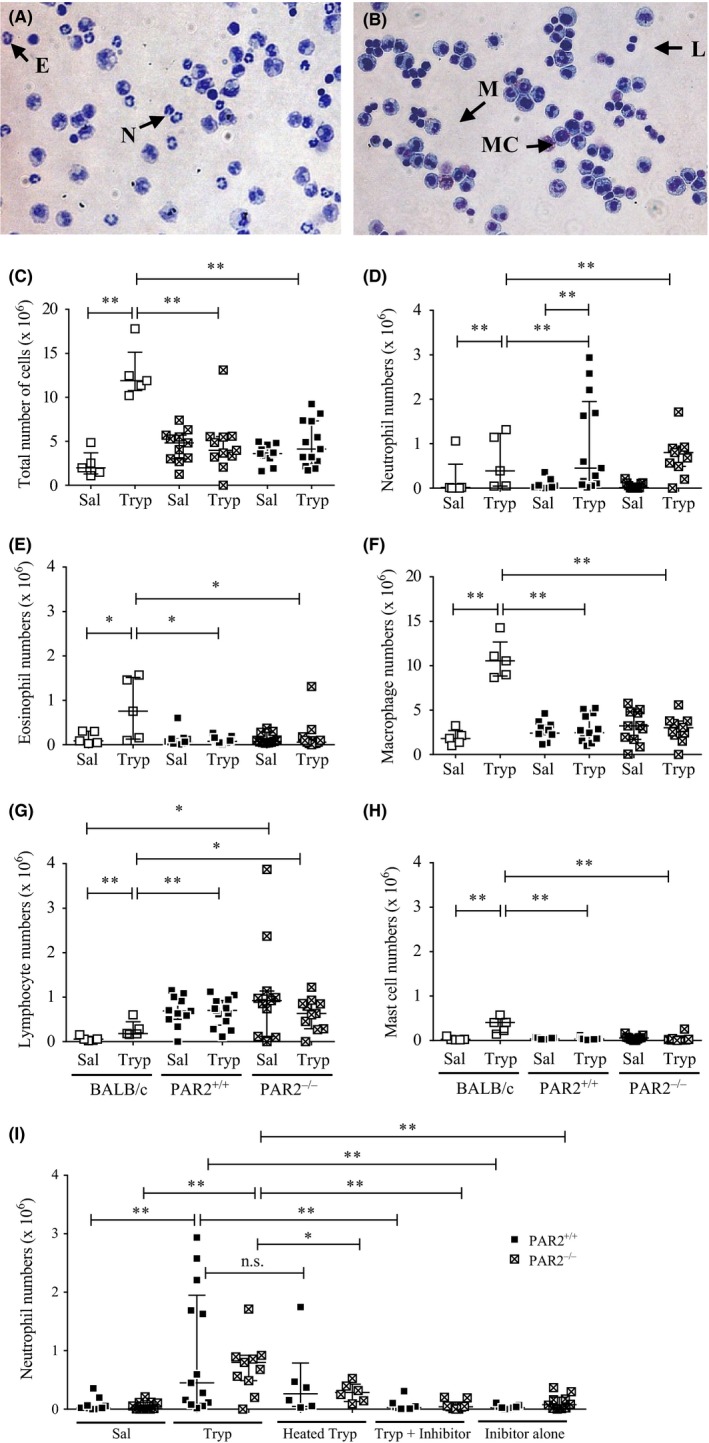
Eosin/methylene blue‐stained cytocentrifuge preparations of cells recovered by peritoneal lavage from C57BL/6 mice 24 hours following injection with (A) tryptase or (B) saline. Examples of neutrophils (N), eosinophils (E), macrophages (M), lymphocytes (L) and mast cells (MC) are indicated. Numbers are indicated of (C) nucleated cells, (D) neutrophils, (E) eosinophils, (F) macrophages, (G) lymphocytes and (H) mast cells recovered from the peritoneum of BALB/c, C57BL/6 PAR2^+/+^ and C57BL/6 PAR2^−/−^ mice 24 hours following injection of tryptase (Tryp; 0.5 μg/mouse). Saline‐injected mice (Sal). Also shown are (I) neutrophil numbers in peritoneal lavage fluid from PAR2^−/−^ and PAR2^+/+^ mice 24 hours following injection of tryptase, heated tryptase, tryptase pre‐incubated with the selective inhibitor, the inhibitor alone and the saline vehicle. N = 5‐13 mice per group. Median values and interquartile range are shown.*P < .05, **P < .005, (Mann‐Whitney *U* test). n.s., not significant

Cells in peritoneal lavage fluid that were recovered from saline‐injected mice were predominantly macrophages, with smaller numbers of lymphocytes and very much smaller numbers of other cell types (Table 1; Figure 1). Injection of tryptase into the peritoneum of wild‐type and PAR2‐deficient mice had little effect on the total number of nucleated cells recovered up to 24 hours afterwards in peritoneal lavage fluid. Tryptase injection was associated with a substantial increase in neutrophil numbers in wild‐type mice at 24 hours (Table 1; Figure 1D). An apparent segregation of wild‐type mice into those with mild (n = 8) or extensive neutrophilia (n = 5) was observed, although neutrophil numbers in each of these subgroups were significantly greater than in the saline‐injected control mice (*P* = .030 and *P* = .0005, respectively; Mann‐Whitney *U* test). Significant neutrophilia was seen also in the PAR2‐deficient mice. There was a trend for increased numbers of neutrophils to be recovered in peritoneum also at 6 and 12 hours, although this reached significance only at 6 hours for the PAR2‐deficient mice (data not shown). Relative numbers of eosinophils, lymphocytes, macrophages and mast cells appeared to be little affected by injection of tryptase at any time‐point. There were no significant differences between these two mouse strains in the numbers of other cells types in the peritoneum.

**Table 1:** Total and differential cell counts in peritoneal lavage fluid recovered from mice 24 hours following injection of saline vehicle, 0.5 μg tryptase, 0.5 μg SLIGRL‐NH_2_ or 0.5 μg LSIGRL‐NH_2_ in wild‐type and PAR2‐deficient mice

Compounds injected	Mouse strain	Total cells	Neutrophils	Eosinophils	Lymphocytes	Macrophages	Mast cells
Saline	PAR2^+/+^	3.60 (3.07‐4.59)	0.02 (0‐0.06)	0.07 (0.03‐0.13)	0.69 (0.50‐1.01)	2.41 (2.24‐3.33)	0.03 (0.02‐0.06)
	PAR2^−/−^	4.81 (3.01‐5.70)	0.01 (0‐0.11)	0.07 (0.05‐0.28)	0.92 (0.12‐1.14)	3.23 (1.71‐4.80)	0.06 (0.02‐0.07)
Tryptase	PAR2^+/+^	4.11 (2.45‐7.32)	0.45**(0.10‐1.95)	0.08 (0.04‐0.33)	0.70 (0.37‐0.92)	2.45 (1.67‐4.49)	0.03 (0.01‐0.05)
	PAR2^−/−^	3.96 (3.21‐5.52)	0.80** (0.49‐0.92)	0.06 (0.03‐0.18)	0.63 (0.29‐0.86)	3.02 (2.21‐3.77)	0.01 (0.01‐0.03)
SLIGRL‐NH_2_	PAR2^+/+^	4.02 (3.33‐4.88)	0.03††(0.01‐0.06)	0.18* ^,^ †† (0.09‐0.43)	0.36† (0.28‐1.09)	2.80 (2.20‐3.85)	0.03 (0‐0.06)
	PAR2^−/−^	4.22 (3.22‐5.67)	0.07††(0.01‐0.28)	0.17 (0.09‐0.32)	0.45 (0.17‐1.99)	3.47 (1.10‐4.83)	0.03† (0.02‐0.10)
LSIGRL‐NH_2_	PAR2^+/+^	3.57 (2.95‐5.18)	0.28** (0.12‐0.48)	0.35** ^,^ †† (0.19‐0.66)	0.19** ^,^ †† (0.14‐0.31)	2.96 (2.19‐3.63)	0.02* (0.01‐0.03)
	PAR2^−/−^	6.31** ^,^ † (5.39‐10.1)	0.02†† (0.01‐0.07)	0.03 (0.02‐0.12)	0.30† (0.20‐0.51)	5.78** ^,^ †† (4.85‐9.17)	0.20** ^,^ † (0.16‐0.21)

Median values (×106) are shown for 10‐12 mice in each group. * *P* < .05 ** *P* < .005, compared with responses in the saline‐injected group; and **^†^** *P* < .05, **^†^^†^** *P* < .005, compared with the responses in the tryptase‐injected group.

As the pattern of cell accumulation in mice of the C57BL/6 strain differed from that reported previously following injection of human lung tryptase in BALB/c mice,^10^ comparison was made between the responses of C57BL/6 and BALB/c mice injected with recombinant tryptase under the same conditions. Injection of tryptase in the peritoneum of BALB/c mice was associated with increases in numbers of neutrophils, eosinophils, lymphocytes, macrophages and mast cells as well as in the total number of cells, compared with those in the saline‐injected group (Figure 1C‐G). This contrasted with the findings in the C57BL/6 mice, in which there were no significant increases in numbers of cells other than neutrophils. Total cell numbers as well as numbers of macrophages, mast cells and eosinophils were all higher in the BALB/c mice when compared with those in the C57BL/6 mice. Although the baseline lymphocyte numbers were lower in BALB/c mice, the degree of tryptase‐induced accumulation of lymphocytes was greater. On the other hand, tryptase did not induce lymphocyte accumulation in C57BL/6.

Heat inactivation of tryptase was associated with lower numbers of neutrophils compared with the tryptase‐injected groups particularly in the PAR2‐deficient mice (Figure 1I). Tryptase‐induced neutrophilia was much less when administered with the selective tryptase inhibitor, and a similar trend was seen with leupeptin (data not shown). The peptide agonist of PAR2 SLIGRL‐NH_2_ failed to replicate the actions of tryptase, and the control peptide employed (LSIGRL‐NH_2_) actually stimulated a small increase in numbers of certain cell types (Table 1).

### 3.2 Effect of tryptase on peritoneal gelatinase and elastolytic activity

**Figure 2:**
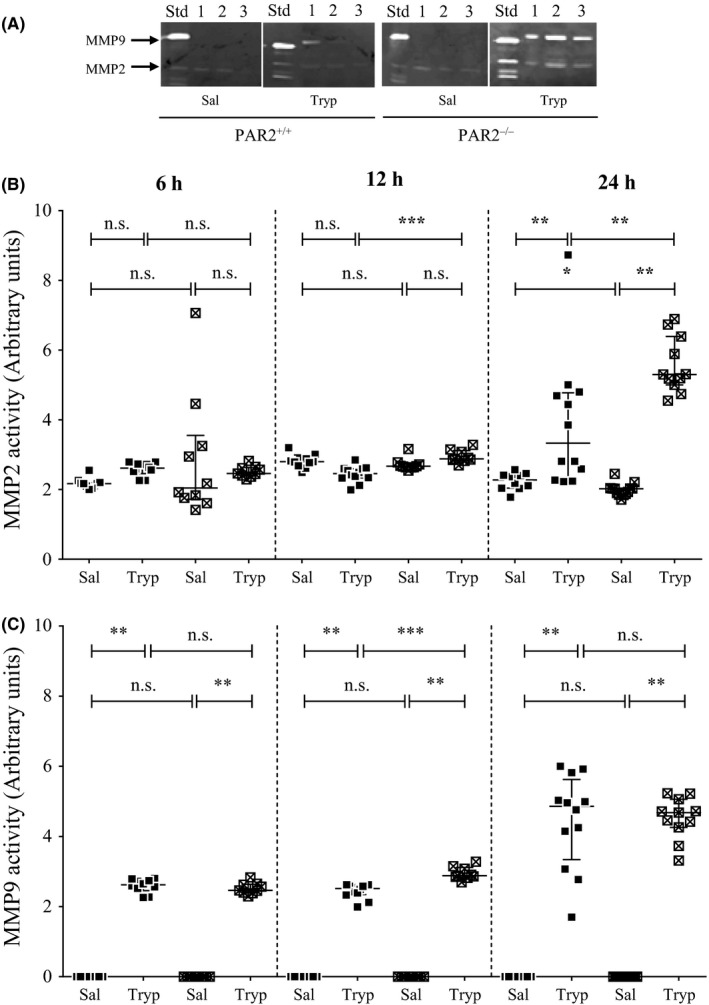
A, Gelatin zymography showing the presence of MMP2 and MMP9 in a standard (Std) supernatant from the HT1080 fibrosarcoma cell line, and supernatants from peritoneal lavage fluid from mice 24 hours after injection of saline alone, or tryptase (mice 1 to 3). B, MMP2 and (C) MMP9 activity in peritoneal lavage fluid from PAR2^−/−^ and PAR2^+/+^ mice 6, 12 and 24 hours following intraperitoneal injection of tryptase (Tryp; 0.5 μg/mouse). Median values and interquartile range are shown. *P < .05 **P < .005 ***P < .0001, (Mann‐Whitney *U* test). n.s., not significant

Peritoneal lavage fluid from saline‐injected mice exhibited activity on gelatin zymography, with bands at positions corresponding to those for MMP2 but not for MMP9 (Figure 2A). Tryptase injection, on the other hand, was associated with the presence of bands in peritoneal lavage fluid for both MMP2 and MMP9. Levels of MMP2 were higher in mice 24 hours following tryptase injection than at 6 and 12 hours and, at this time‐point, were significantly greater than in the saline‐injected mice (Figure 2B). At all of these time‐points, MMP9 activity was substantially greater in the tryptase‐injected than the saline‐injected mice, although levels at 24 hours were greater than at 6 or 12 hours (Figure 2C).

Peritoneal lavage fluid MMP2 activity was higher in tryptase‐injected mice deficient in PAR2 than in the corresponding wild‐type mice at 12 hours (*P* < .0001) and 24 hours (*P* < .005), but not at 6 hours (Figure 2B). For the saline‐injected mice, there were no apparent strain‐related differences in MMP2 levels. For MMP9 activity, there was greater activity in the PAR2 knockout mice than in the wild‐type at 12 hours (*P* < .005), but not at 6 or 24 hours. Mice injected with heat‐inactivated tryptase did not show a marked tryptase‐induced increase in MMP2 and MMP9 activity in peritoneal lavage fluid (Table 2). Similarly, incubation of tryptase with the selective inhibitor appeared to abolish the ability of tryptase to stimulate an increase in MMP2 and MMP9 activity.

**Table 2:** MMP2 and MMP9 activity (Arbitrary units), albumin levels (mg mL^−1^), total protein concentration (μg mL^−1^) and histamine levels (ng mL^−1^) in peritoneal lavage fluid from mice 24 hours following injection of the saline vehicle, tryptase, heated tryptase, tryptase pre‐incubated with the selective inhibitor and the inhibitor alone in PAR2^+/+^ and PAR2^−/−^ mice

	Mouse strain	Saline	Tryptase alone	Heated tryptase	Inhibitor alone	Tryptase + inhibitor
MMP2	PAR2^+/+^	2.2 (2.0‐2.4)	3.3** (2.4‐4.8)	2.4* ^,^ † (2.2‐2.5)	1.9†† (1.8‐2.2)	1.9†† (1.7‐2.5)
	PAR2^−/−^	2.0 (1.9‐2.0)	5.3** (5.0‐6.4)	2.3†† (2.1‐2.3)	2.1†† (1.9‐2.3)	1.7* ^,^ †† (1.7‐1.8)
MMP9	PAR2^+/+^	ND	4.9** (3.3‐5.6)	ND	ND	ND
	PAR2^−/−^	ND	4.7** (4.3‐5.1)	ND	ND	ND
Albumin	PAR2^+/+^	2.5 (2.0‐2.8)	8.0** (5.4‐11)	8.9** (8.2‐9.0)	12†† (11‐13)	8.0** (6.1‐8.6)
	PAR2^−/−^	2.1 (1.8‐2.7)	11** (8.6‐12)	8.0** ^,^ † (8.2‐8.4)	11 (3.7‐12)	8.5** ^,^ † (9.0‐11)
Total protein	PAR2^+/+^	510 (395‐547)	200** (54.0‐368)	319* (193‐520)	393* ^,^ † (361‐442)	524†† (439‐611)
	PAR2^−/−^	389 (315‐460)	541 (244‐906)	528* (455‐628)	484 (368‐580)	283* (242‐‐322)
Histamine	PAR2^+/+^	133 (79‐159)	54.5** (44.5‐99.0)	115 (49.2‐253)	–	–
	PAR2^−/−^	254 (126‐268)	47.8** (16.8‐80.9)	265†† (233‐269)	–	–

Median and interquartile range values are shown for 10‐12 mice each group. *P < .05 **P < .005 ***P < .0001, compared with response in the saline‐injected group. ^†^ *P* < .05 ^††^ *P* < .005, compared with response in the tryptase‐injected group. ND, none detected; ‐, not tested.

In peritoneal lavage fluid, levels of MMP2 and MMP9 activity were closely correlated in both tryptase‐injected wild‐type mice (*r*_s_ = .882, n = 33, *P* < .005) and PAR2‐deficient mice (*r*_s_ = .975, n = 30 *P* < .005). The activity of MMP2 was associated with the numbers of neutrophils recovered from the peritoneum of wild‐type (*r*_s_ = .335, n = 33, *P* < .005) and knockout mice (*r*_s_ = .328, n = 30, *P* < .005). Similarly, the activity of MMP9 was correlated with the number of neutrophils from both wild‐type (*r*_s_ = .452, n = 33, *P* < .005) and knockout mice (*r*_s_ = .360, n = 30, *P* < .005). Associations were not observed between MMP2 or MMP9 levels and the numbers of any of the other cell types enumerated. With elastase as a positive control in elastin zymography at quantities as low as 4.5 μg, there were bands at 28 and 22 kDa (data not shown). However, supernatants from peritoneal lavage fluid from mice of both the saline and tryptase‐treated groups failed to show any activity.

Injecting the PAR2 agonist SLIGRL‐NH_2_ into either mouse strain failed to reproduce the substantial increases in MMP activity induced by tryptase. In wild‐type mice, MMP2 activity recovered in peritoneal lavage fluid 24 hours following injection of SLIGRL‐NH_2_ (2.33, 2.21‐2.47 units; median, interquartile range) did not differ from that of those injected with saline (2.28, 2.04‐2.42 units; Mann‐Whitney *U* test), and in the corresponding experiment with PAR2‐deficient mice, MMP2 activities were slightly higher in mice injected with the PAR2 agonist (2.08, 2.06‐2.14 units) than in those with saline (2.00, 1.86‐2.04 units; *P* = .01). An increase in MMP2 activity was not observed under similar conditions following injection of the non‐PAR2‐activating peptide LSIGRL‐NH_2_ in either the wild‐type (1.74, 1.68‐1.86) or PAR2‐deficient mice (2.11, 2.00‐2.30) and, in fact, that in the wild‐type mice was actually lower than in saline‐injected mice (*P* = .001). No MMP9 activity was found in any of these mice following the injection of SLIGRL‐NH_2_, LSIGRL‐NH_2_ or saline.

### 3.3 Identification of neutrophils as source of gelatinase activity

**Figure 3:**
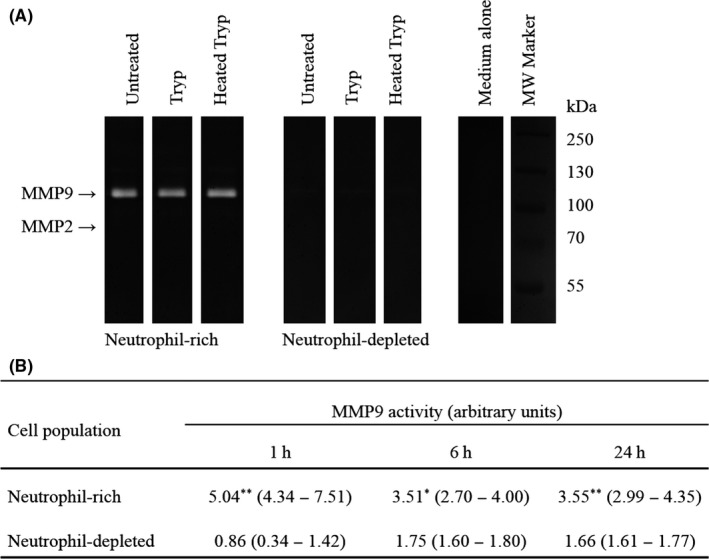
A, Gelatin zymography illustrated for neutrophil‐rich and neutrophil‐depleted peritoneal lavage cell populations isolated from 8 mice 24 hours following tryptase injection. Culture supernatants were incubated for 1 hours with tryptase at 13 μg mL^−1^ (40 mU mL^−1^), heat‐inactivated tryptase (of the same original concentration) or medium alone. Bands for molecular weight (MW) markers are also shown. B, Relative MMP9 activity in culture supernatants of neutrophil‐rich and neutrophil‐depleted cell populations maintained in culture for 1, 6 or 24 hours (n = 6 for each group). Median and interquartile range are indicated. *P < .05, **P < .005, (Mann‐Whitney *U* test) compared with activity in the neutrophil‐depleted cell population

Supernatant from a neutrophil‐rich cell population (88% neutrophils) obtained from the peritoneum of 10 tryptase‐injected PAR2 wild‐type mice (by positive selection with magnetic beads) had substantially more MMP9 than that from a neutrophil‐depleted cell population (12% neutrophils; Figure 3). This was observed when cells were incubated in vitro for 1, 6 and 24 hours. There was little MMP2 activity in supernatants of peritoneal cells at any of these time‐points. The supernatant of a mixed cell population from casein‐injected mice (n = 5) also showed MMP9 activity but that from naïve mice (n = 3) had negligible quantities (data not shown).

Treatment of either neutrophil‐rich or neutrophil‐depleted cell populations with 6.5 or 13 μg mL^−1^ (20 or 40 mU mL^−1^) tryptase in vitro was not associated with alterations in levels of MMP9 in cell culture supernatants (Figure 3B). The release of MMP9 into cell supernatants thus appeared to be constitutive. As found for peritoneal lavage fluid, no elastase activity was detected by elastin zymography in supernatants from cultured neutrophil‐rich or neutrophil‐depleted cell populations.

### 3.4 Albumin, total protein and histamine levels in peritoneal lavage fluid

The albumin concentration in peritoneal lavage fluid was greater in tryptase‐injected mice at 24 hours compared with those injected with saline (Table 2). Apparent increase in albumin levels was induced with inactivated tryptase by heating or the selective inhibitor. Differences were not observed between wild‐type and PAR2 knockout mice in the concentrations of albumin in lavage fluid. At 6 hours or 12 hours following injection of tryptase, the albumin levels in the peritoneal lavage fluid were not significantly different from those in the saline‐injected mice (data not shown). There was no apparent association between levels of albumin with either histamine concentrations in peritoneal lavage or with numbers of mast cells recovered.

The total protein concentration in peritoneal lavage fluid as detected by BCA binding did not differ between mice injected with tryptase and PAR2 agonist at 24 hours or at 6 hours or 12 hours (data not shown). There was no association between concentrations of albumin and total protein levels in mouse peritoneal lavage fluid. For both wild‐type and PAR2‐deficient mice injected with tryptase, at 24 hours, peritoneal lavage fluid histamine levels were lower than in the saline‐injected mice. This effect was not seen with heated tryptase or at the earlier time‐points with catalytically active tryptase.

## 4 DISCUSSION

The present study confirms and extends the idea that mast cell tryptase has potent pro‐inflammatory actions, but suggests that the effects are not mediated through activation of PAR2. Tryptase was a potent stimulus for the accumulation of neutrophils in vivo and induced MMP release and prolonged microvascular leakage. Despite several studies suggesting that the actions of tryptase are mediated by cleavage of PAR2, at least in the present model, the role tryptase as a mediator of inflammation appears to be independent of PAR2.

Care was taken to ensure that the tryptase employed in this study was of high purity and activity, and on SDS‐PAGE, recombinant tryptase appeared as a single band whose identity was confirmed by Western blotting. The endotoxin levels were extremely low, and our finding that heating the tryptase at 90°C for 20 minutes abolished its proteolytic activity would argue against a role for endotoxin in the inflammatory changes provoked. The finding that the β‐tryptase is a potent stimulus for recruitment of neutrophils in vivo provided confirmatory evidence that tryptase can induce massive neutrophilia in the mouse peritoneum.^10^ It is possible, therefore, that β‐tryptase represents the variant of tryptase primarily responsible for this action.

This observation of tryptase‐induced increases in the numbers of eosinophils, macrophages and lymphocytes as well as of neutrophils (at both 6 hours and 16 hours) in the peritoneum of BALB/c mice 10 differed from that of the present study in which we have used C57BL/6 mice (selected on account of the background of the PAR2‐deficient mice). However, the recruitment of eosinophils and macrophages, and even of mast cells (a cell type whose presence was not investigated previously), was induced in the present studies by intraperitoneal injection of BALB/c mice with β‐tryptase. The discrepancy between studies is thus likely to be related to the mouse strain rather than to the type of tryptase‐injected. HayGlass et al have reported a little difference between BALB/c and C57BL/6 in the degree of neutrophilia or eosinophilia when these mice were immunized with Der p1, ovalbumin or human serum albumin.^48^ On the other hand, Th1 responses have been reported to be more prominent in C57BL/6 mice and Th2 responses to be stronger in BALB/c mice infected with Leishmania.^49^ Strain‐related differences in the present study are likely to be related to the nature of the inflammatory infiltrate, as adoptive transfer of Th2 cells has been reported to stimulate eosinophilia in a mouse model of ovalbumin challenge, but neutrophilia in mice reconstituted with Th1 cells.^50^

Injection of tryptase into the mouse peritoneum was associated with high levels of albumin in peritoneal lavage fluid 24 hours after of injection, indicating increased microvascular permeability. Intradermal injection of tryptase has been reported previously to induce microvascular leakage and oedema in the skin of guinea‐pigs or sheep 20 minutes following injection.^12^, ^13^ The skin reactions were prolonged and still apparent 60‐80 min following injection, but at least in the guinea‐pig model had resolved by 6 hours. Microvascular leakage was attributed to the ability of tryptase to stimulate release of histamine through activation of mast cells, and this has been demonstrated experimentally in vitro with guinea‐pig tissue and subsequently also in human mast cells.^37^ A role for mast cells in the microvascular leakage provoked as late as 24 hours after tryptase injection in the present study cannot be excluded, but albumin concentrations were not associated with numbers of mast cell or concentrations of histamine, and levels of histamine were actually lower in mice injected with tryptase than in the saline‐injected control mice.

Being present in such increased numbers, the neutrophil deserves consideration as a contributor to the microvascular leakage observed, and this cell is, like the mast cell, an important source of proteases. Neutrophil elastase has been reported to induce an increase in the permeability of monolayers of epithelial cell lines,^51^, ^52^ although, in the present study, elastase could not be detected in the peritoneal lavage fluid by elastin zymography. Stronger evidence in the present model was provided for the presence of neutrophil‐derived MMP9 which has also been shown to be implicated in stimulating microvascular leakage.^53^ The tryptase‐induced increases in the number of neutrophils were highly significantly correlated with MMP9 levels in the peritoneal lavage fluid at 24 hours, and production of this gelatinase was observed in vitro in short‐term cultures of peritoneal cells enriched for neutrophils but not in neutrophil‐depleted cells. Much of the gelatinase activity determined in the peritoneal lavage fluid is likely to have been derived from neutrophils either directly, or possibly indirectly through the release of proteases which can cleave and activate both MMPs derived from other cell types.^54^ Naïve macrophages, as well as B and T lymphocytes, have also been implicated as sources of MMP2 and MMP9,^55^, ^56^ albeit in very small quantities as MMP9 has not previously been detected in peritoneal lavage fluid from C57BL/6 mice.^57^, ^58^

Heating tryptase or pre‐incubating this protease with the selective tryptase inhibitor effectively inhibited tryptase‐induced neutrophilia dependence on an intact catalytic site but not the tryptase‐induced microvascular leakage. The latter finding may reflect involvement of a non‐proteolytic mechanism for tryptase as has been reported previously,^59^ or possibly a lack of potency for the inhibitor (which itself stimulated microvascular leakage at the concentration employed). However, the present studies strongly suggest that PAR2 is not a key substrate for tryptase in this model. Patterns of tryptase‐induced inflammatory cell accumulation were similar for both wild‐type and PAR2‐deficient mice. The PAR2 knockout mice had higher peritoneal lavage levels of MMP2 at 12 and 24 hours, and of MMP9 at 12 hours compared to the wild‐type mice, but the differences were relatively small; and there were no differences between these mouse strains in relative concentrations of albumin, histamine or total protein. Intraperitoneal injection of the PAR2 peptide agonist SLIGRL‐NH_2_ did not stimulate altered numbers of cells in the peritoneal lavage fluid of mice or changes in levels of gelatinases or any of the other markers examined as did tryptase.

The PAR2 agonist peptide was able to provoke eosinophilia in wild‐type (but not in the PAR2‐deficient mice), an observation consistent with a report of eosinophil accumulation following intrapleural administration of this peptide in BALB/c mice.^60^ However, in our model, injection of LSIGRL‐NH_2_, a control peptide without demonstrable actions on PAR2,^61^ was also able to stimulate cell accumulation. Biological actions have been noted previously with this peptide, including altered flux of rolling leucocytes in naive rats after superfusion 22 and also calcium mobilization in cultured human endothelial cells (Khedr et al, unpublished data).

Recruitment of polymorphonuclear leucocytes was found following injection of SLIGRL‐NH_2_ into the peritoneum of rats,^22^ but as in our study, it has been reported that this PAR2 agonist did not induce an inflammatory infiltrate when administered into the peritoneum of BALB/c mice.^23^, ^62^ Similarly, administration of SLIGRL‐NH_2_ into mouse airways failed to induce neutrophilia, and this peptide was found to actually inhibit cell accumulation provoked by LPS.^62^ While the greater susceptibility to degradation may be a factor, there is thus little evidence to indicate that SLIGRL‐NH_2_ can reproduce the actions of tryptase.

The similarity in tryptase‐induced inflammatory changes in wild‐type and PAR2‐deficient mice argues against PAR2 having a key role in mediating the actions of tryptase. The potential of tryptase to activate this receptor has been questioned previously, with Huang et al failing to observe PAR2 activation in vitro following addition of tryptase to cells.^11^ The lack of PAR2 activation was attributed to functional heterogeneity in tryptases, and it was suggested that there may be different substrate specificities between the βI‐tryptase they employed and the βII‐tryptase or lung‐derived tryptase that had been employed previously by others to activate PAR2.^1^, ^2^, ^3^, ^20^ However, the variant of tryptase employed in the present studies (βII‐tryptase) is the same as that described as being able to activate PAR2.^38^, ^63^ The tetrameric structure of tryptase with the catalytic sites positioned within a central pore^64^ is likely to restrict access of the extracellular domain of PAR2 as a substrate, and heavy glycosylation of the N‐terminal sequence of PAR2 has been reported to prevent tryptase‐induced receptor activation.^8^ Moreover, tryptase can cleave PAR2 not just at a site that would result in exposure of the tethered ligand, but also at a point that could “disarm” the receptor.^2^

The non‐PAR2‐mediated processes involved in the present model remain open to conjecture. Certain other proteases have been shown to alter cell behaviour through regulation of growth factor receptors. Thus, hormone‐like cellular signalling has been described through the actions of tryptic proteases on receptors themselves (as with insulin receptors or insulin‐like growth factor‐1 receptors),^65^ and the release of a membrane tethered agonist, for example, heparin‐binding epidermal growth factor (EGF) by metalloproteinases.^66^ In theory, also a growth factor agonist could be generated from precursors through proteolytic activity. The ability of tryptase to control the bioavailability of cytokines has been suggested on the basis of the finding that this protease can activate TGFβ, and at least in mice, to inactivate IL‐6 from mast cells.^28^, ^67^ Unravelling the processes involved will represent a major challenge.

In conclusion, our findings indicate that tryptase is a potent stimulus of inflammation in vivo, and as such deserves attention as a target for therapeutic intervention. At quite low concentrations, this protease can stimulate neutrophilia, microvascular leakage and the generation of MMP9 and MMP2. The studies with PAR2 knockout mice challenge the assumption that activation PAR2 represents the primary mechanism by which tryptase can mediate inflammatory changes. The actions of tryptase were dependent on an intact catalytic site, but in the model employed, the pro‐inflammatory actions of tryptase appear to be largely independent of PAR2.

## CONFLICT OF INTEREST

The authors declare no conflict of interest.

## AUTHOR CONTRIBUTIONS

MMS Khedr and AM Abdelmotelb were responsible for data acquisition and analysis. AF Walls, X Zhou and SLF Pender contributed to the conception and design of the work or of parts of it and to its interpretation. MMS Khedr and AF Walls drafted and revised the manuscript. AM Abdelmotelb, SLF Pender and X Zhou revised it critically for intellectual content.
